# Genetic diversity trend in Indian rice varieties: an analysis using SSR markers

**DOI:** 10.1186/s12863-016-0437-7

**Published:** 2016-09-05

**Authors:** Nivedita Singh, Debjani Roy Choudhury, Gunjan Tiwari, Amit Kumar Singh, Sundeep Kumar, Kalyani Srinivasan, R. K. Tyagi, A. D. Sharma, N. K. Singh, Rakesh Singh

**Affiliations:** 1 Division of Genomic Resources, ICAR-National Bureau of Plant Genetic Resources, New Delhi, 110 012 India; 2 Division of Germplasm Conservation, ICAR-National Bureau of Plant Genetic Resources, New Delhi, 110 012 India; 3 ICAR-National Research Centre on Plant Biotechnology, Pusa Campus, IARI, New Delhi, 110012 India

**Keywords:** Rice, HvSSR markers, Hierarchical analysis, Model based analysis, Decadal analysis, India

## Abstract

**Background:**

The knowledge of the extent and pattern of diversity in the crop species is a prerequisite for any crop improvement as it helps breeders in deciding suitable breeding strategies for their future improvement. Rice is the main staple crop in India with the large number of varieties released every year. Studies based on the small set of rice genotypes have reported a loss in genetic diversity especially after green revolution. However, a detailed study of the trend of diversity in Indian rice varieties is lacking. SSR markers have proven to be a marker of choice for studying the genetic diversity. Therefore, the present study was undertaken with the aim to characterize and assess trends of genetic diversity in a large set of Indian rice varieties (released between 1940–2013), conserved in the National Gene Bank of India using SSR markers.

**Result:**

A set of 729 Indian rice varieties were genotyped using 36 HvSSR markers to assess the genetic diversity and genetic relationship. A total of 112 alleles was amplified with an average of 3.11 alleles per locus with mean Polymorphic Information Content (PIC) value of 0.29. Cluster analysis grouped these varieties into two clusters whereas the model based population structure divided them into three populations. AMOVA study based on hierarchical cluster and model based approach showed 3 % and 11 % variation between the populations, respectively. Decadal analysis for gene diversity and PIC showed increasing trend from 1940 to 2005, thereafter values for both the parameters showed decreasing trend between years 2006-2013. In contrast to this, allele number demonstrated increasing trend in these varieties released and notified between1940 to 1985, it remained nearly constant during 1986 to 2005 and again showed an increasing trend.

**Conclusion:**

Our results demonstrated that the Indian rice varieties harbors huge amount of genetic diversity. However, the trait based improvement program in the last decades forced breeders to rely on few parents, which resulted in loss of gene diversity during 2006 to 2013. The present study indicates the need for broadening the genetic base of Indian rice varieties through the use of diverse parents in the current breeding program.

**Electronic supplementary material:**

The online version of this article (doi:10.1186/s12863-016-0437-7) contains supplementary material, which is available to authorized users.

## Background

Rice (*Oryza sativa* L.) is a staple food crop in India and many parts of the world. In India, it occupies the largest area under cultivation and has maximum share in grain production [3]. India is one of the centers for rice diversity and large diversity has been reported both at inter- and intra- specific levels [36]. Yield, quality characters and tolerance to biotic and abiotic stresses are major objectives of varietal development [25].

A large number of rice varieties are released and notified every year in India with higher yields, tolerance to biotic and abiotic stresses and to meet the requirement of changing farming systems based on user demands. Different rice varieties of distinct genetic background are a good promise for the future rice crop improvement. This has contributed to a large extent to the major increases in agricultural productivity in the twentieth century [10]. It is generally thought that continuous selection among the crosses of genetically related cultivars has led to a narrowing of the genetic base of the crops on which modern agriculture is based, thus contributing to the genetic erosion of the crop gene pools [33].

A robust and reliable method of fingerprinting is required for identification and purity testing of these varieties [41], as well as to study the genetic relationships among different cultivars [18, 42]. Genetic characterization of crop plants has gained momentum with the advent of PCR based molecular markers. Nowadays, SSR is a marker of choice for molecular characterization as it is co-dominant, distributed throughout the genome, highly reproducible, variable, reliable, easily scorable, abundant and multi-allelic in nature [37]. SSR markers have been used by many researchers [9, 17, 44] for characterization of rice varieties. SSR markers even in less number can give a better genetic diversity spectrum due to their multi allelic and highly polymorphic nature [24].

Recent reports suggest that genetic diversity in crop varieties released over the years fluctuates in successive time periods [8, 48]. In case of wheat there are reports available which showed an increase [16], decrease [12] as well as constant gene diversity over a period of time [15, 35]. The similar trend was also reported in rice [23, 47]. Over the last few centuries, rice has faced diversity loss [6] especially, after the green revolution due to replacement of native varieties with high yielding varieties [14]. Despite of a large number of varieties being developed in every year, molecular studies on a small set of rice varieties has revealed narrow genetic base [26, 45].

The present study was undertaken with the aim to assess the trend in genetic diversity of Indian rice varieties released and notified over the period from 1940 to 2013 and to understand the genetic relationship amongst the varieties by employing both hierarchical and model based approach using hyper variable simple sequence repeats (HvSSR) markers.

## Results and Discussions

Our study is the first major effort to analyze the trend of genetic diversity in the large set of Indian rice varieties released over the years. A total of 729 varieties released from the year 1940 to 2013 was analyzed using HvSSR markers. These varieties possessed various agronomical and economically important traits such as tolerance to biotic and abiotic stresses (drought, cold, salinity and lodging), aroma content, grain yield and early maturity etc. (Additional file 1: Table S1).

### HvSSR marker based analysis

Thirty-six HvSSR markers were used to characterize 729 rice varieties. Gene diversity, heterozygosity, major allele frequency and PIC were calculated for all the 36 HvSSR markers. A total of 112 alleles was amplified with an average of 3.11 alleles per locus (Table 1). Similar observations were also reported; 3.02 alleles per locus with SSR markers during characterization of 25 Indian rice hybrids [2] and 3 alleles per locus in a set of 192 Indian rice germplasm characterization [25]. The number of alleles amplified per HvSSR primers varied from 2 to 5 with maximum numbers of alleles (5) being amplified by primers; HvSSR09-11, HvSSR11-21, HvSSR11-58 and HvSSR12-13. A similar number of alleles (2–5) for SSR markers were reported in 141 Basmati rice accessions of North Western Himalaya [37]. The PIC values for HvSSR primers ranged from 0.04 (HvSSR06-16) to 0.58 (HvSSR03-37) with a mean of 0.29. Shah et al. [38] and Pachauri et al. [29] have reported mean PIC values 0.37 and 0.38, respectively in different sets of rice varieties which were closer to our result. On the other hand, Pal et al. [30] reported mean PIC value 0.40 on a set of basmati and non-basmati varieties and Salgotra et al. [37] have reported mean PIC value 0.40 in a basmati collection of north-western Himalaya, which were little higher than our result. The gene diversity ranged from 0.04 (HvSSR06-16) to 0.66 (HvSSR03-37) with an average of 0.33. Gene diversity obtained in the present study was quite low as compared to 0.52 [25] and 0.54 [6] reported in rice germplasm lines and varieties, respectively. Heterozygosity varied from 0.92 (HvSSR03-02) to 0.00 (HvSSR05-30) with an average of just 0.15. The low level of heterozygosity has also been reported in other studies on rice [5, 25] and this could be attributed to its self pollination behavior. The major allele frequency was also calculated for all 36 HvSSR markers which ranged from 0.37 (HvSSR03-37) to 0.97 (HvSSR06-16) with an average of 0.76 (Table 1). The average major allele frequency in the present study was higher as compared to the previous studies on Indian rice varieties [46] and Korean landraces [19].

**Table 1:** List of HvSSR primers used for genotyping of 729 rice varieties along with their chromosomal position, product size, No. of alleles amplified, gene diversity, heterozygosity and PIC value

Chr. No.	Primer ID	Size (bp)	Forward primer	Reverse primer	Major allele frequency	Allele no	Gene diversity	Heterozygosity	PIC
1	HvSSR01-32	250	AAACTGGAGATGAACTCGAA	GTAACGAACTAGAGCATGGG	0.9445	2.0000	0.1048	0.0340	0.0993
	HvSSR01-41	348	TGAGTGAGACTTGACAGTGC	AGTTAACACCAATGCTGACC	0.5943	3.0000	0.5405	0.2340	0.4638
	HvSSR01-53	274	TGTCGTCCACGTAGTAGGAG	ACACTCCTCCTCTGTTCTCA	0.8894	2.0000	0.1968	0.0030	0.1774
2	HvSSR02-01	312	AAGAGATGAGAAGAGCAATGA	CAACTTAGAGGAAGAAGGAGG	0.7813	2.0000	0.3417	0.0892	0.2834
	HvSSR02-33	355	TAATGCACGCACAACTTTAC	TATAGAATGCTGACTGGGCT	0.7976	2.0000	0.3228	0.0923	0.2707
	HvSSR02-50	195	TTTCAGGAATCTGATGCTTT	TTAATCAAAGCCCTAACAGC	0.6579	3.0000	0.5077	0.1445	0.4547
3	HvSSR03-02	228	TAGCGGAGTTGGAATAACAC	CTGCACTGCATACCTCATAA	0.5346	4.0000	0.5737	0.9224	0.4922
	HvSSR03-10	280	GTACACAACGTCACAACAGC	ACTGTGGCATATGTTCGATT	0.7566	2.0000	0.3683	0.1468	0.3005
	HvSSR03-19	230	AATTCAGTTCACGCATTCTT	AGCTGTTCGTCTGCATAGTT	0.8968	2.0000	0.1851	0.0577	0.1680
	HvSSR03-37	386	GGAAATCGTCAAGAACGTC	TAATTGTATACCACTCCGCC	0.3780	3.0000	0.6610	0.3013	0.5868
	HvSSR03-54	352	GCCTATCAGGCTATCATCAC	GTGATCGACATTGAGGAGTT	0.8168	2.0000	0.2993	0.0049	0.2545
4	HvSSR04-19	265	TCGTGGAGTATCCTGTATCC	TTATAACTTGGAGCTCAGGC	0.9127	3.0000	0.1631	0.1041	0.1566
	HvSSR04-27	318	ATGGATTTAGGCTTGTTTGA	ATACTGCGAAGGTGAAGAGA	0.7150	4.0000	0.4609	0.2629	0.4319
	HvSSR04-46	179	GGCGCGCTTATATATGTACT	CGATTGCGTGGTGTAACTAT	0.7774	4.0000	0.3712	0.1475	0.3413
5	HvSSR05-09	335	CTCTCCATCTTGCAATCTTC	TGCATGACTCTATCAACCAG	0.5331	3.0000	0.5509	0.0768	0.4558
	HvSSR05-15	275	CCATGTCAAACGGTTACTTT	GGGAGAAGTGAGAAAGAGGT	0.9366	3.0000	0.1205	0.0975	0.1166
	HvSSR05-30	353	TACGACGGACGATTAAAGTT	GCTAACTCATTCATCTCGCT	0.7962	2.0000	0.3245	0.0000	0.2719
6	HvSSR06-03	212	CTAGGGAATCAGCGGTTAG	GCTCTCTTGTCCTTCTTCTTC	0.9582	3.0000	0.0802	0.0099	0.0771
	HvSSR06-16	368	TCTGAAATGCTGTCATCAAG	GAGCAGAGTAGGACATGAGC	0.9767	2.0000	0.0454	0.0186	0.0444
	HvSSR06-40	385	CTCTTCCGTGGTTAAAGAAA	CACTGGTATGATCTCCGACT	0.5405	3.0000	0.5285	0.2859	0.4232
7	HvSSR07-18	343	GGTGTGTTGTCGAATCTCTC	ATGCCATTGTCCTTACATTC	0.5436	3.0000	0.5383	0.1317	0.4392
	HvSSR07-51	341	CGAGCATGTCTGTCAAGTAA	GTTCGAATGTAATGTTGGCT	0.8503	3.0000	0.2645	0.0349	0.2464
8	HvSSR08-14	295	TCCACTTTACATCGTCACAA	CTACCTCTTAACCGCACATT	0.5996	3.0000	0.5401	0.0736	0.4661
	HvSSR08-19	221	CATCTCTTGAGAAATCTGCC	TGTGCATTTCGTCTTTCATA	0.4944	4.0000	0.5183	0.1278	0.4022
9	HvSSR09-11	366	TGCAGAATTTCTTCCTTCAT	ACCAGAATCTCCCAAATGTA	0.7632	5.0000	0.3981	0.0371	0.3752
	HvSSR09-26	331	TGGGCATCTGGTACTATCTT	AGCTCATTCCACAGGTTAGA	0.9594	2.0000	0.0779	0.0056	0.0749
	HvSSR09-55	382	TTACTCCGCATATATCCATGT	ATTTGACACCAAGTTGATCC	0.5925	4.0000	0.5090	0.7326	0.4105
10	HvSSR10-03	289	TCTTTCCCAAATTCCAGATA	CATTAGTTGTTTGTGGCAGA	0.9081	3.0000	0.1711	0.0883	0.1641
	HvSSR10-13	169	CAGGGAATCAACATCAAAGT	AGCAAGGCAAGTCATCTCTA	0.5795	4.0000	0.5292	0.4003	0.4378
	HvSSR10-34	202	TAGACCGAGGAATTGAAAGA	TTTGGGCTTATTGTCAGTTT	0.9060	4.0000	0.1749	0.0982	0.1681
11	HvSSR11-13	285	TGAAACCACAATGAGTCAAA	GCCCTAAACCCAAATAGAAG	0.7532	2.0000	0.3718	0.1062	0.3027
	HvSSR11-21	291	TACGCTATAACCATGAAGCA	CTCCCGTTATTGTCCTTACA	0.8305	5.0000	0.2962	0.2161	0.2766
	HvSSR11-58	371	ACTGAATCCTTACTGGAGCA	GGAGATAAGCATTTGGAAGA	0.7881	5.0000	0.3639	0.1607	0.3451
12	HvSSR12-01	271	GATTTGCAACACGTACGATA	GATCATCCACTCTGAGCAAT	0.9203	2.0000	0.1467	0.0986	0.1360
	HvSSR12-13	388	ACCTTAGGGCTGAGTTCTTT	TTAGGCTTGTCTCTTCCTCA	0.9048	5.0000	0.1782	0.0710	0.1733
	HvSSR12-39	289	ATCTAACAACAACAATCCCG	CATCTTCATCCCTCGTGTAT	0.8793	4.0000	0.2211	0.1142	0.2124
	Mean				0.7630	3.1111	0.3346	0.1536	0.2917

### Hierarchical cluster analysis

**Fig. 1:**
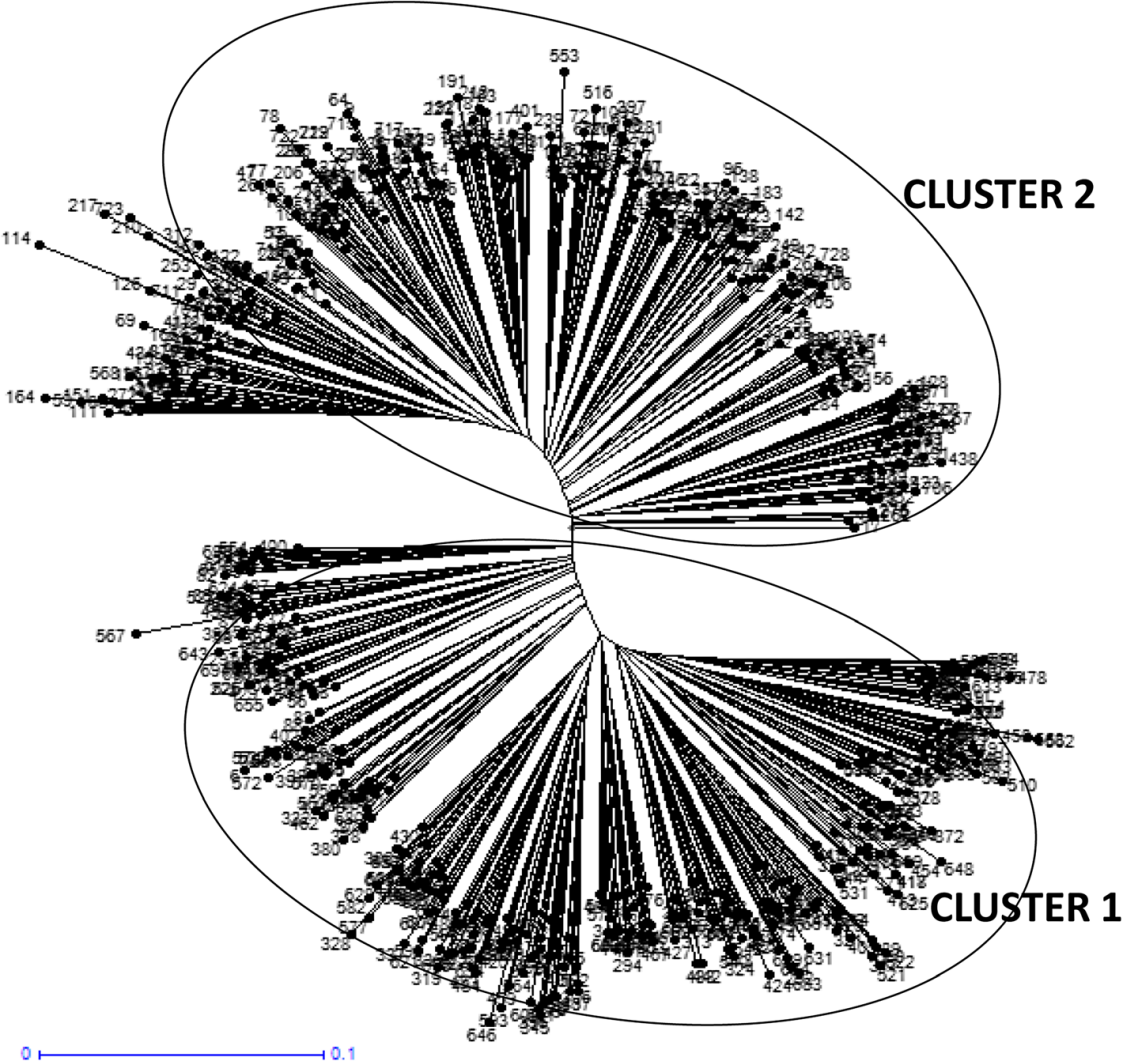
NJ tree of the 729 rice varieties

The amplicons generated by HvSSR markers across 729 varieties were used for cluster development using the neighbour joining (NJ) method. The unrooted tree (Fig. 1) grouped 729 rice varieties into two major clusters, 400 varieties in cluster1 whereas; 329 varieties were grouped in cluster2. This grouping was further supported by studies of Upadhyaya et al. [46], Nachimuthu et al. [25] and Das et al. [9] who also have reported two clusters during their studies in Indian rice germplasm. Further, we also analyzed clustering pattern of rice varieties based on their traits. Passport data of some of the varieties contained information about a few key traits which helped us in studying trait based grouping in the NJ clusters. Out of 40 aromatic rice varieties, 25 were grouped into cluster2 and 15 into cluster1. Similarly, out of 15 salt tolerant varieties nine were present in cluster2 and six were present in the cluster1. This suggests that for the development of aroma and salinity tolerant varieties breeders may have used diverse parents, as reflected by their grouping in both the clusters. Additionally, we also analyzed clustering pattern of hybrids. Interestingly, out of 30 hybrid varieties studied in the set, 22 (73.3 %) were grouped into cluster2 and just eight in cluster1. In cluster1 out of eight hybrids six were having IR series parents common (Additional file 2: Table S2).

### Model based population structure

**Fig. 2:**
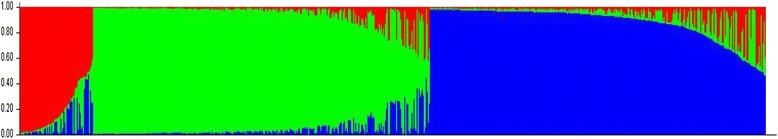
Population structure of 729 rice varieties

**Fig. 3:**
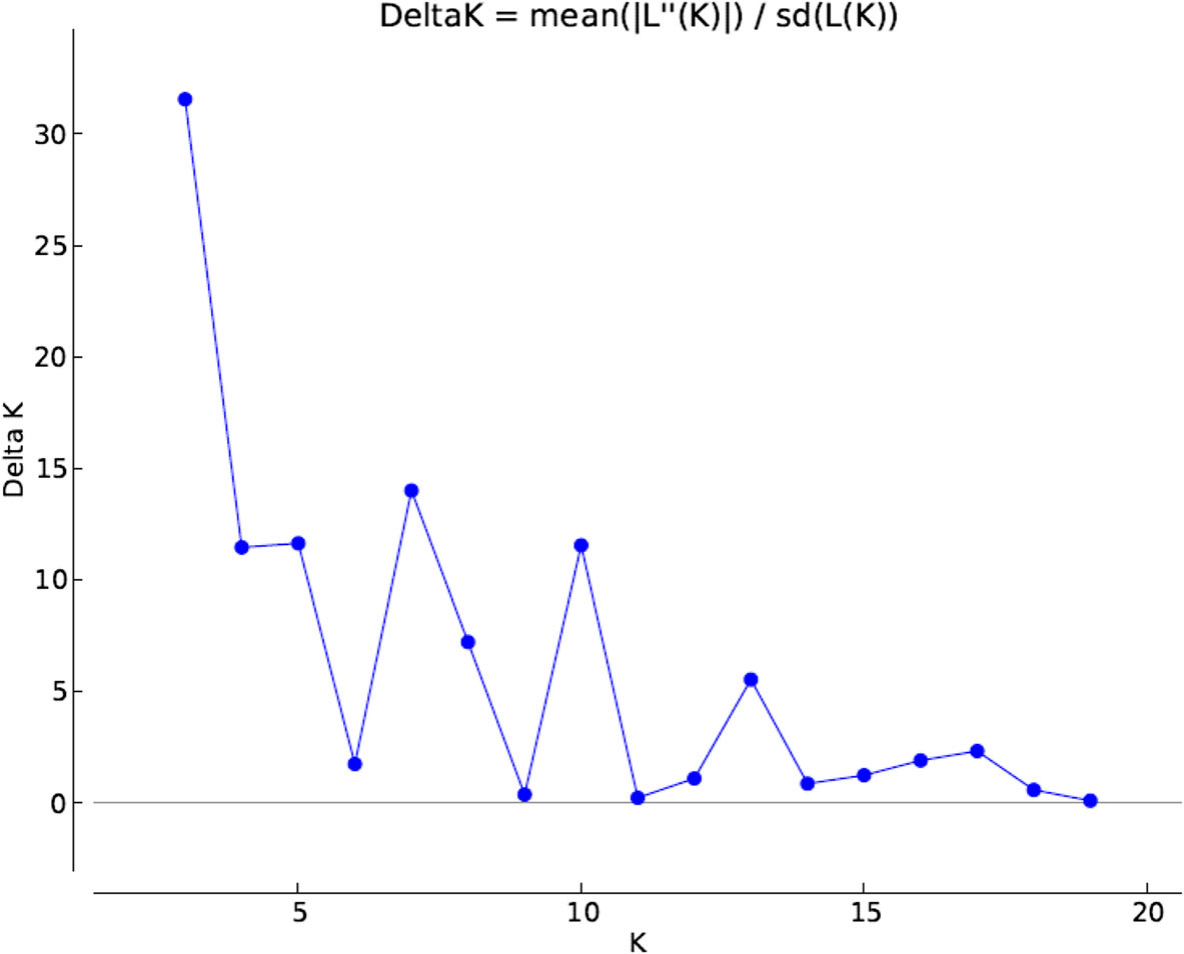
Estimation of population using LnP(D) derived Δk for k from 1 to20

Population structure divided 729 varieties into 3 populations (Figs. 2 and 3 and Additional file 3: Figure S1). Population1 (pop1), population2 (pop2) and population3 (pop3) contained 72, 329 and 328 varieties, respectively. Further, based on the membership fractions, varieties under different populations were categorized as pure or admixture. The varieties with the probability more than ≥0.80 score was considered as pure and less than 0.80 as an admixture. Pop1 showed 44 pure (61 %) and 28 admixed (38 %) individuals, pop2 showed 282 pure (86 %) and 47 (14 %) admixed individuals and pop3 showed 260 pure (79 %) and 68 (20 %) admixed individuals. The mean Fsts value of pop1, pop2 and pop3 were 0.0118, 0.3240 and 0.2667, respectively, and mean alpha value was 0.0829 (Table 2). The allele frequencies (divergence among populations) were 0.0686 between pop1 and pop2; 0.0533 between pop1 and pop3 and 0.0548 between pop2 and pop3 (Table 3). Earlier studies on population structure have reported two to eight sub-populations using different rice collections [1, 4, 13, 20, 49–51]. Roy et al. [36] and Upadhyaya et al. [46] have also reported similar population number in different set of Indian rice varieties. The relatively small value of alpha (α = 0.0829) in present study reveals that, only few individuals were admixed. Alpha value approaching zero indicates that most individuals in the study are from separate populations [19] whereas; an alpha value greater than 1 indicates that most of accessions of populations are admixed [28]. Distributions of rice varieties in different populations based on their traits were also studied. All 30 hybrid varieties and most of the aromatic rice varieties were grouped in pop2 (Fig. 2 and Additional file 3: Figure S1). It was also observed that 190 varieties out of 329 in pop2 were released after 1979, which indicates that most of the recently released varieties were present into pop2. Both hierarchical and model based population structure showed that large number varieties in cluster2 (347) and pop2 (329) correspond to each other.

**Table 2:** Mean value of alpha, Fst1, Fst2 and Fst3 inferred from model based approach

Mean	Value
Mean value of alpha	0.0829
Mean value of Fst_1	0.0118
Mean value of Fst_2	0.324
Mean value of Fst_3	0.2667

**Table 3:** Allele-frequency divergence among populations computed using estimates of P (Model based approach)

	Pop1	Pop2	Pop3
Pop1	0	0.0686	0.0533
Pop2	0.0686	0	0.0548
Pop3	0.0533	0.0548	0

### AMOVA and PCoA of clusters obtained using hierarchical approach

**Fig. 4:**
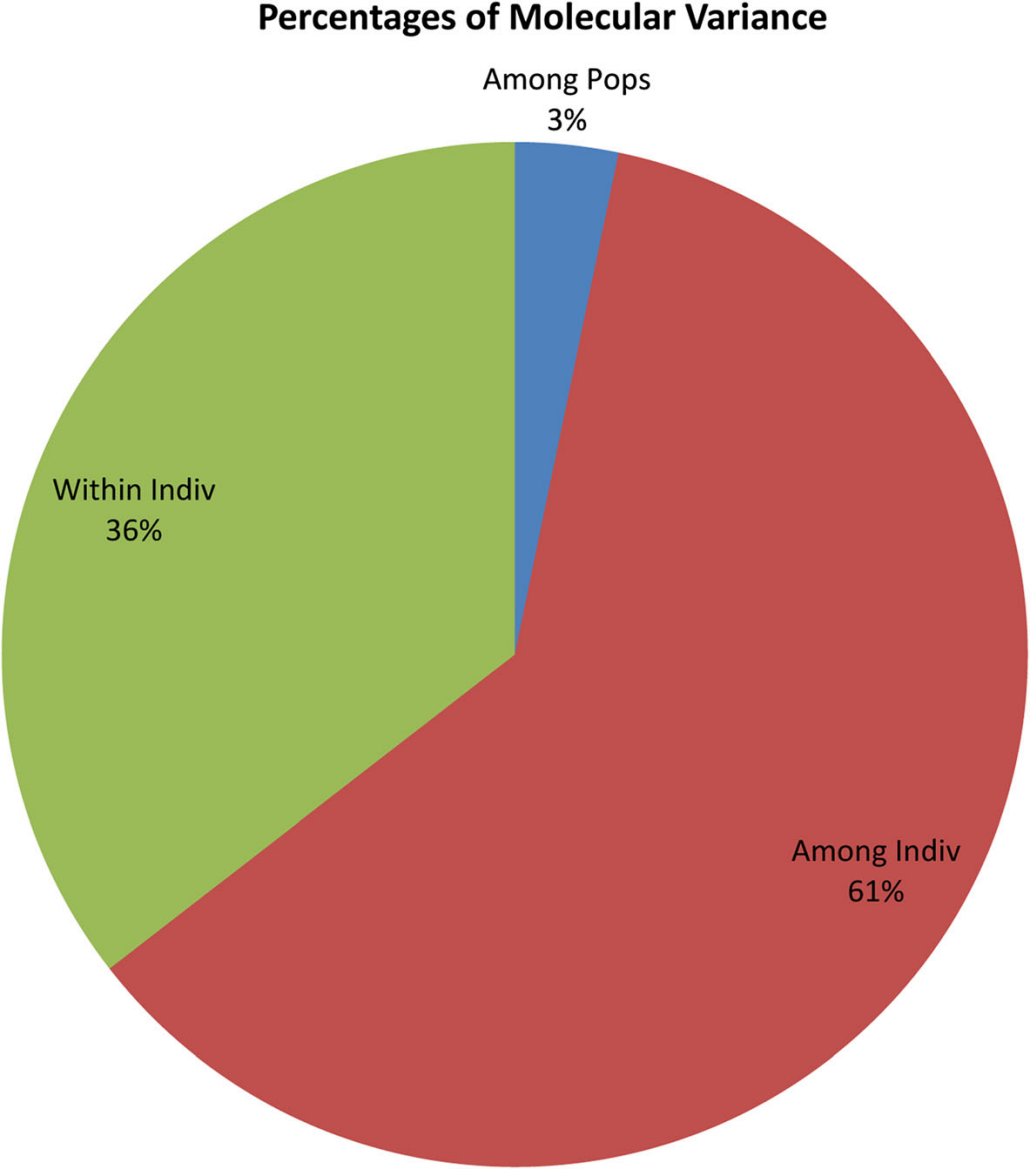
Analysis of molecular variance (AMOVA) of 729 rice varieties based on populations obtained by hierarchical approach

**Fig. 5:**
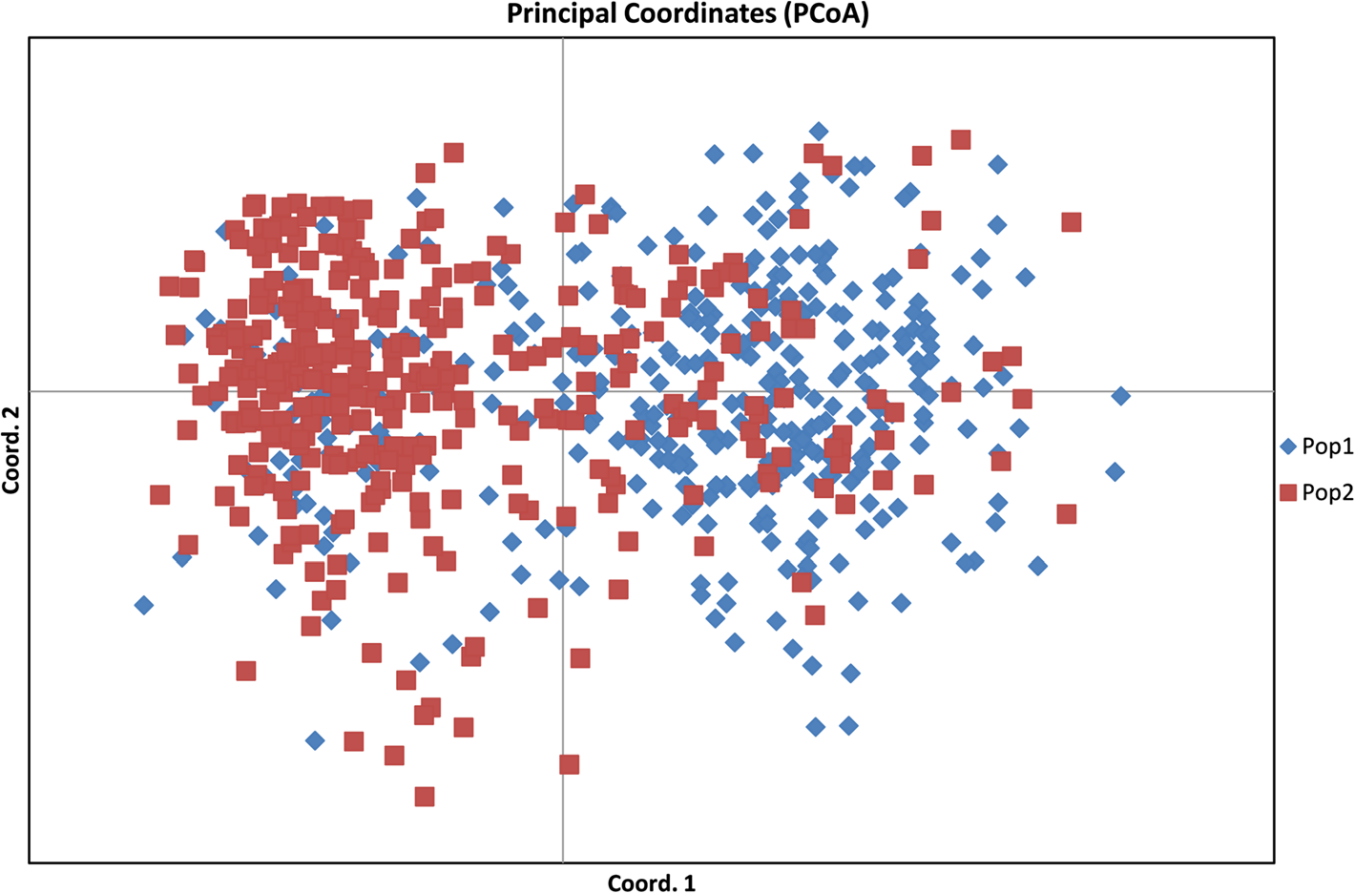
Principal coordinate analysis (PCoA) of 729 rice varieties based on populations obtained by hierarchical approach

AMOVA for the 729 varieties was performed based on the two clusters obtained using hierarchical cluster analysis. The two populations showed 3 % variance among themselves, whereas, 61 % variance was recorded among individuals and 36 % variance within individuals (Fig. 4 and Table 4). PCoA based on hierarchical clusters (labeled with two different colours) showed intermixing of two groups across the coordinates (Fig. 5). The first three axes explained 15.9 % of cumulative variation (Table 5).

**Table 4:** Summary of analysis of molecular variance (AMOVA) for hierarchical clustering approach

Source	Df	SS	MS	Est. Var.	%
Among Pops	1	187.128	187.128	0.241	3 %
Among Indiv	726	8517.340	11.732	4.548	61 %
Within Indiv	728	1919.000	2.636	2.636	35 %
Total	1455	10623.468		7.425	100 %

**Table 5:** Percentage of variation explained by the first 3 axes using Principal coordinate analysis for hierarchical clustering approach

Axis	1	2	3
%	8.08	4.14	3.66
Cum %	8.08	12.23	15.89

### AMOVA and PCoA of populations obtained using model based approach

**Fig. 6:**
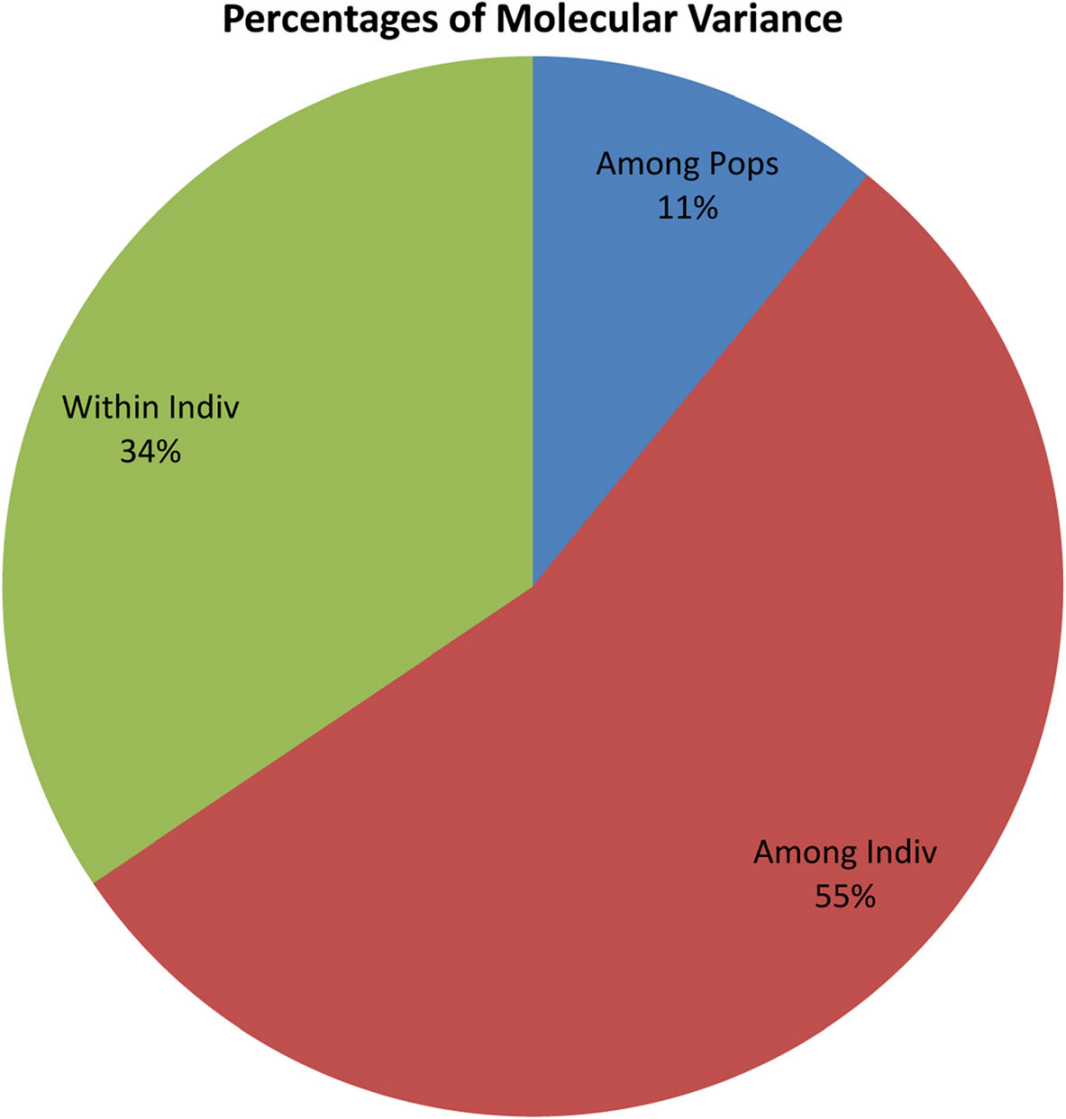
Analysis of molecular variance (AMOVA) of 729 rice varieties based on population obtained by model based approach

**Fig. 7:**
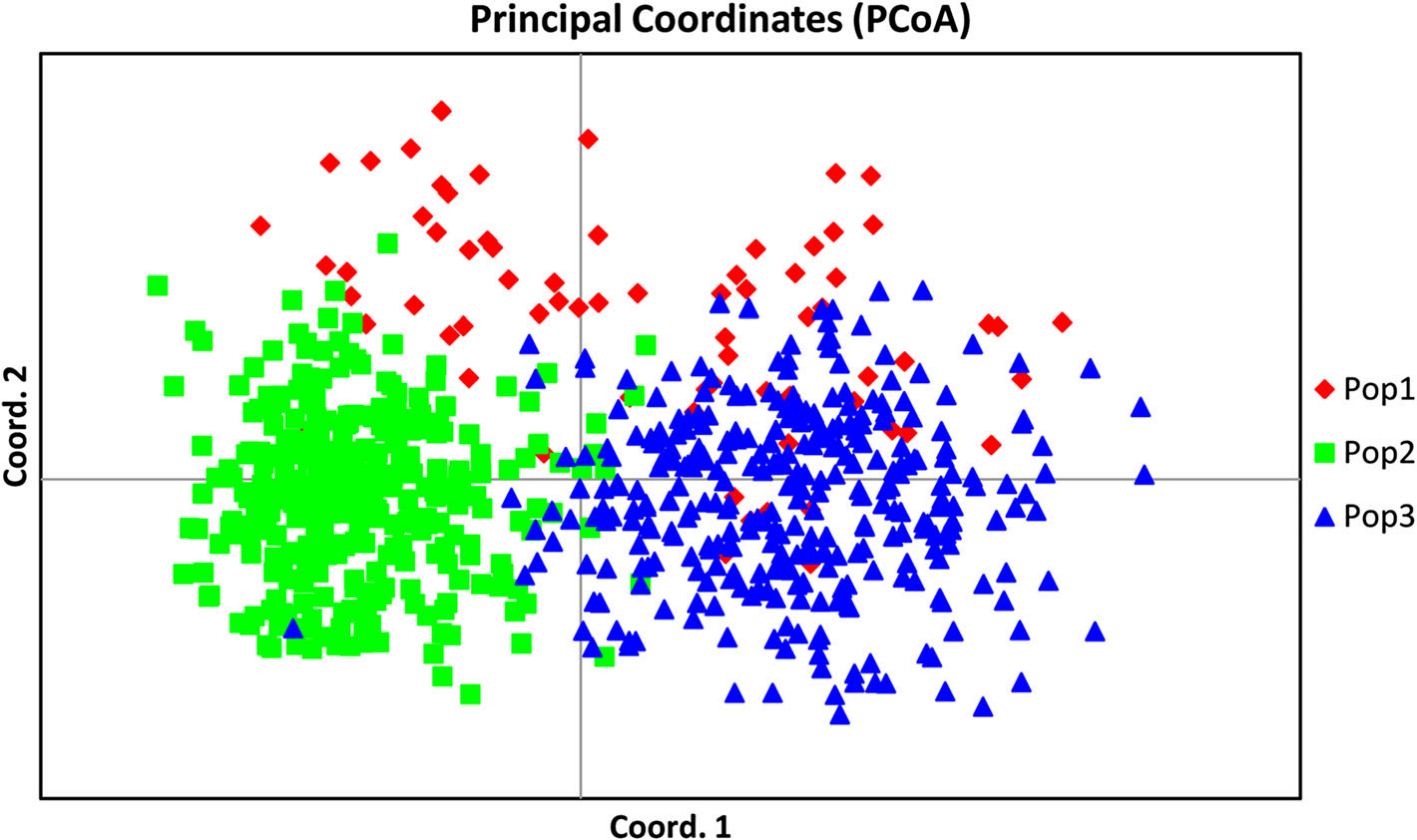
Principal coordinate analysis (PCoA) of 729 rice varieties based on population obtained by model based approach

AMOVA was performed on three populations obtained using a model based approach. Among three populations 11 % variance was recorded, whereas, among individuals, 55 % variance and within individuals, 34 % variance was found (Fig. 6 and Table 6). Choudhury et al. [7] have also reported similar pattern of variation in Indian rice germplasm using populations derived from the model based approach. PcoA revealed that large genetic diversity exists in Indian rice varieties. The first three axes explained 15.9 % of cumulative variation (Table 7). In PcoA, rice varieties were labeled with three different colours which represent the three populations obtained from population structure (Fig. 7). The pop1 and pop2 showed distinct grouping whereas; the individuals of pop3 were distributed over pop1 and pop2.

**Table 6:** Summary of analysis of molecular variance (AMOVA) for model based approach

Source	df	SS	MS	Est. Var.	%
Among Pops	2	729.778	364.889	0.831	11 %
Among Indiv	726	7985.068	10.999	4.182	55 %
Within Indiv	729	1921.000	2.635	2.635	34 %
Total	1457	10635.846		7.648	100 %

**Table 7:** Percentage of variation explained by the first 3 axes using Principal Coordinate analysis for model based approach

Axis	1	2	3
%	8.08	4.14	3.66
Cum %	8.08	12.23	15.89

Hierarchical based AMOVA analysis showed less (3 %) variation among population, compared to model based structure population (11 %). The reason for less variation between populations in case of hierarchical clusters may be due to the number of groups predicted (two clusters) which was higher in case of model based approach (three groups).

### Decadal genetic diversity trend analysis in Indian rice varieties

To understand the genetic diversity trend in Indian rice varieties released and notified from 1940 to 2013 an interval of 10-years was taken to keep the number of varieties almost constant in each interval. Except for interval 1940–1965, which was about 26 years, other intervals were about 10 years. Decadal analysis showed a steady increase in gene diversity of varieties released during 1966–1975 (0.314), 1976–1985 (0.315), 1986–1995 (0.335), 1996–2005 (0.346) but it showed decreasing trend during 2006–2013 (0.290). In contrast to gene diversity, major allele frequency showed decreasing trend in the varieties released during 1940 (0.774) to 2005 (0.751) whereas; it showed an increase during 2006–2013 (Table 8). Number of alleles (Na) increased remarkably up to 17.3 % in the varieties if we compare the allele number present in the interval 1940–1965 with the allele number present in the interval 1966–1975. But after 1975, only 3.9 % increase was observed, when interval of 1966–1975 was compared with an interval of 1976–1985 and 1 % increase in the number of alleles was recorded when 1976–1985 interval was compared with 1986–1995. Allele number was constant during 1986–1995 to 1996–2005 interval, but it showed 3 % increase from interval 1996–2005 to 2006–2013. Similar trends in the number of alleles (Na) was reported by Choudhary et al. [6], whereas; in contrast to this Wei et al. [47] reported decreasing trend in Na after 1980s using different set of rice collections. Similarly, gene diversity also showed a steady increase in the varieties released during 1940s to 2005, but it decreased in those released during 2006–2013. Mantegazza et al. [23] have reported steady increase in the gene diversity level in Italian rice germplasm; in contrary to this no change was observed in the level of diversity in rice varieties of Nepal [43]. PIC also showed increasing trend in all varieties released over the period of 1940 (0.267) to 2005 (0.302) whereas; it showed a decrease during 2006–2013 (0.255). Comparison of per cent increase in PIC value showed an increase of 2.27 % between interval 1940–1965 and interval 1966–1975, but no major change was recorded for the interval 1966–1975 and interval 1976–1985. Further, it showed an increase of 7.13 % and 3.45 % between interval 1976–1985 and 1986–1995 and between intervals 1986–1995 and 1996–2005, respectively. But it drastically decreased (15 %) between 1996–2005 and 2006–2013. It clearly indicates massive loss of genetic diversity in the last 10 years, which may be attributed to shifting towards the trait specific breeding during this period. According to Choudhary et al. [6], the reason for this trend could be the selection priorities of breeders for need based breeding.

**Table 8:** Pattern of gene diversity, heterozygosity, major allele frequency and PIC over decadel periods

Intervals	Major allele frequency	Genotype no	Allele no	Gene diversity	Heterozygosity	PIC
1940–1965	0.7745	2.8056	2.4167	0.3172	0.2000	0.2676
1966–1975	0.7835	3.7222	2.8333	0.3148	0.1550	0.2737
1976–1985	0.7767	4.4444	2.9444	0.3155	0.1467	0.2732
1986–1995	0.7588	4.6667	2.9722	0.3352	0.1390	0.2927
1996–2005	0.7515	4.5278	2.9167	0.3468	0.1568	0.3028
2006–2013	0.8032	4.5556	3.0000	0.2909	0.1566	0.2553

### Pedigree-based analysis of hierarchical cluster and model based population

Analysis of varieties sharing common parentage (Additional file 1: Table S1) showed that they were grouped in the same cluster (Fig. 1) or population (Fig. 2). For example, varieties sharing common parentage like CNM-25 and CNM-31, GR11 and GR4, ADT 31 and ADT 33, Kumbham and Makaram were grouped into common cluster2 and pop2. Similarly, Archana and Pusa 4-1-11, KalingaI and KalingaII, Vjaya and Jayanthi were grouped into pop2 and cluster1. Chaitanya and Krishnaveni, Deepti and Nandi, Aruna and Remya, PTB-39 and PTB-41, Sasyasree and Vikas, Moniram, Bahadur, Piolee and Kushal were grouped into pop3 and cluster1, Dharitri and Savitri were grouped into pop3 and cluster2 (Figs. 1 and 2 and Table 9). There were a few exceptions where varieties having common parentage were not grouped together in the same cluster or population. For example, Kanchi and Vaigai were grouped into same population (pop2) but, in different clusters. Similar trend in pedigree based clustering was also observed by Upadhyay et al. [6, 46]. Upadhyay et al. [46] showed that varieties with at least one common parent were grouped in one cluster and Choudhary et al. [6] showed that varieties released during different decades were also grouped together due to the presence of common parents in their pedigree.

**Table 9:** Rice varieties sharing common parentage

Name of the variety	Year of release/notification	Common parents	Cluster	Population
CNM-25	1980	IR-8	2	2
CNM-31	1980		2	2
GR-11	1977	Z-31 and IR-8-246	2	2
GR-4	1981		2	2
IR-22	1978	IR 8 and Tadukan	2	2
IR-579	1975		2	3
ADT 31	1975	IR 8 and Cul. 340	2	2
ADT 33	1979		2	2
Kanchi	1970	TN 1 and CO 29	2	2
Vaigai CO 37	1974		1	2
Kumbham (KTR- 3)	1998	Cherady	2	2
Makaram (KTR- 2)	1998		2	2
Archana	1974	IR8 and Tadukan	1	2
Pusa - 4-1- 11			1	2
Kalinga II	1973	Dunghansalia and IR8	1	2
Kalinga I	1973		1	2
Vijaya	1970	T90 and IR8	1	2
Jayanthi (AC-30919)	1973		1	2
Chaitanya	1989	Sowbhagya and ARC 5984	1	3
Krishnaveni	1990		1	3
Nandi (MTU-5182)	1993	Sowbhagya and ARC-6650	1	3
Deepti (MTU-4870)	1997		1	3
Aruna (MO-8)	1989	Jaya and PTB 33	1	3
Remya (MO-10)	1989		1	3
PTB-39	1972	PTB 10 and IR-8	1	3
PTB-41	1972		1	3
Krishna Anjana (MO-19)	2002	MO-1 and MO-6	1	3
Karishma (MO-18)	1998		1	1
Moniram	1991	Pankaj and Mahsuri	1	3
Piolee	1991		1	3
Bahadur	1994		1	3
Kushal	1994		1	3
Ranjit	1994		2	3
Ratna	1970	TKM-6 and IR-8	1	3
Sasya Sree	1980		1	3
Vikas	1983		1	3
Dharitiri	1989	Pankaj and Jagannath	2	3
Savitri (Ponmani)	1983		2	3

### Co-linearity between hierarchical cluster and model based population analysis

**Fig. 8:**
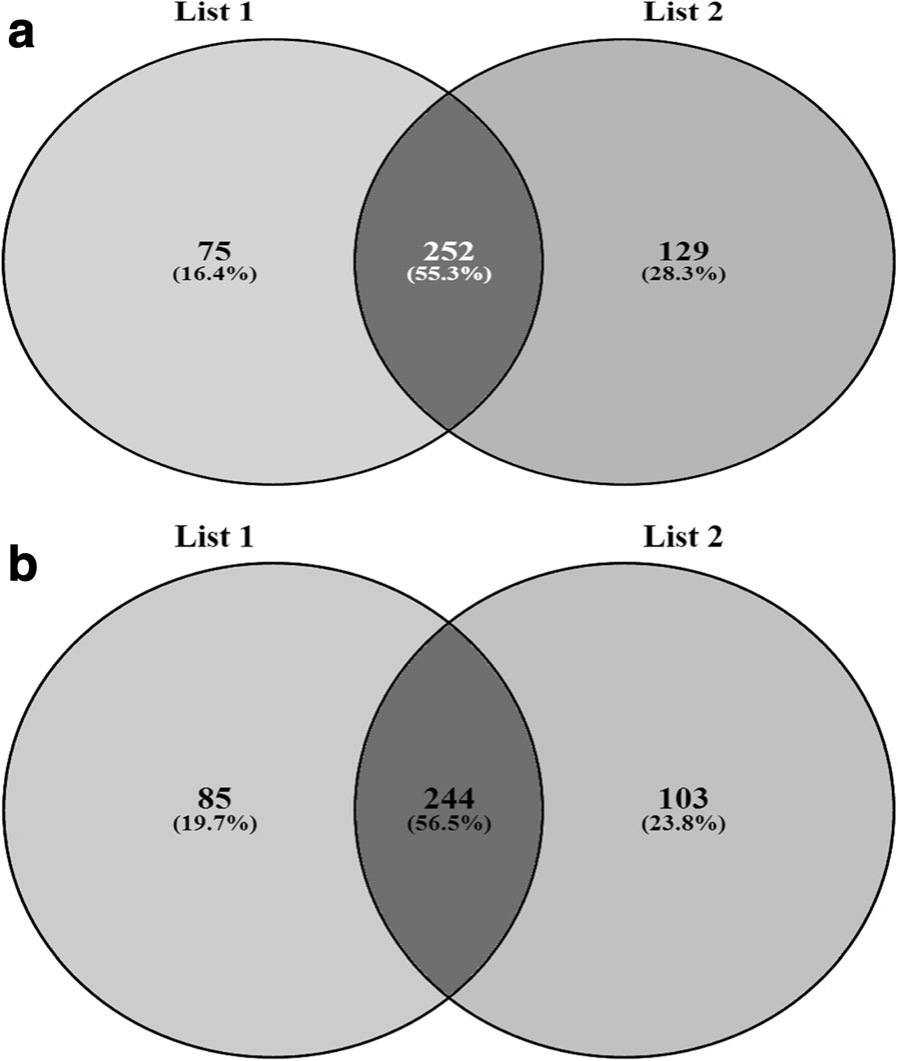
**a** Venn diagram showing co linearity between population 2 & cluster 2. **b** Venn diagram showing co linearity between population3 & cluster1

The Co-linearity between varieties grouping in hierarchical cluster and model based population structure was confirmed by Venn diagram. Liu et al. [22] studied the Chinese wild rice collection and has also shown that Venn diagram is a robust method to study overlapping accessions. In the Venn diagram out of 729 varieties, 244 rice varieties (56.5 %) were common between pop2 and cluster2. Similarly, 252 rice varieties (55.3 %) were common between pop3 and cluster1 (Fig. 8a and b). This study supports that grouping of rice varieties based on hierarchical cluster and model based approach were more than 55 % similar.

## Conclusion

The present study based on 36 HvSSR markers distributed over all 12 chromosomes of rice suggests that after green revolution breeders have used different parentage for improving the yield, quality and plant architecture, but after 2006 priority of breeders have changed and instead of plant architecture, more focus was on breeding for biotic and abiotic stress tolerance and trait-specific improvement. This could be the possible reason that allele number recorded over the period has not decreased, but genetic diversity and PIC have shown a sudden decrease after 2006. To broaden the genetic base, there is an urgent need to incorporate more diverse donor parents in the breeding program for varietal improvement in rice.

## Methods

### Plant materials

Seed samples of 729 varieties of rice received from Indian National Genebank, ICAR- National Bureau of Plant Genetic Resources (NBPGR), New Delhi. The details of each variety along with passport data (National ID, i.e. Indigenous Collection (IC) number, state, local name, pedigree, traits) are given in Additional file 1: Table S1 [39].

### DNA extraction from rice seed

Seeds of each variety (10–12 seeds) were dehusked and used for DNA isolation using QIAGEN DNeasy plant mini kit (Hilden, Germany). Fine powder was obtained by grinding kernels using tissue lyser (Tissue lyser II Retsch, Germany) with a tissue lyser adapter set (QIAGENq). QIAGEN DNeasy plant mini kit protocol was followed for DNA isolation.

### Genotyping of rice varieties using SSR markers

Initial screening was done with 120 highly variable SSR (HvSSR) marker loci with repeat lengths of 51–70 bp which were located across all 12 chromosomes of rice [40]. Finally 36 most polymorphic markers (3markers/chromosome) were selected, which were covering both long and short arm of rice chromosome for genotyping of 729 rice varieties. Temperature of amplification for each primer was standardized by gradient PCR with selected rice samples.

Working stocks (10 ng/μl) of genomic DNA of all the 729 varieties were prepared. PCR reaction mixture (total volume of 10 μl) contained 2 μl genomic DNA (10 ng/μl), 0.8 μl of 25 mM MgCl_2_, 1 μl of 10X buffer, 0.2 μl of each primer (10 nmol), 0.2 μl of 10 mM dNTPs, 0.2 μl of Taq DNA polymerase (Fermentas, Life Sciences, USA) and 5.6 μl distilled water. The conditions for PCR amplification were as follows: initial denaturation at 94 °C for 4 min followed by 36 cycles of 94 °C for 30 s, Ta for 45 s, 72 °C for 1 min and final extension at 72 °C for 10 min. 4 % metaphor agarose gel was used for analyzing the amplified products with the constant supply of 120 V for 4 h. Gel documentation system (Alpha Imager®, USA) was used to record the gel pictures.

### Statistical analyses

Power Marker 3.5 [21] was used to calculate major allele frequency, gene diversity, heterozygosity and polymorphic information content (PIC) for each locus of HvSSR markers. The genetic distance calculated for each variety with Power marker, which was used for cluster development using neighbor-joining (NJ) tree. The un-weighted neighbor joining tree was constructed using DARwin software 5.0.158 [32] GenAlEx V6.5 [31] was used to study PCoA and AMOVA. To study the population structure model-based program, STRUCTURE 2.3.3 [34] was used and three replications were run for each K. Each run was implemented with a burn-in period of 100,000 steps with 100,000 Monte Carlo Markov Chain replicates [34]. The membership of each genotype was run for a range of genetic clusters from value of K = 1 to 20 by taking the admixture model and correlated allele frequency into account. The plateau of ΔK was obtained by plotting LnPD values derived for each K [11]. The final population was determined using “Structure harvester” program (http://taylor0.biology.ucla.edu/structureHarvester/). Venn diagram analysis was performed to identify common varieties between cluster and population using software Venny 2.1 [27].

### Ten-year interval analysis

**Fig. 9:**
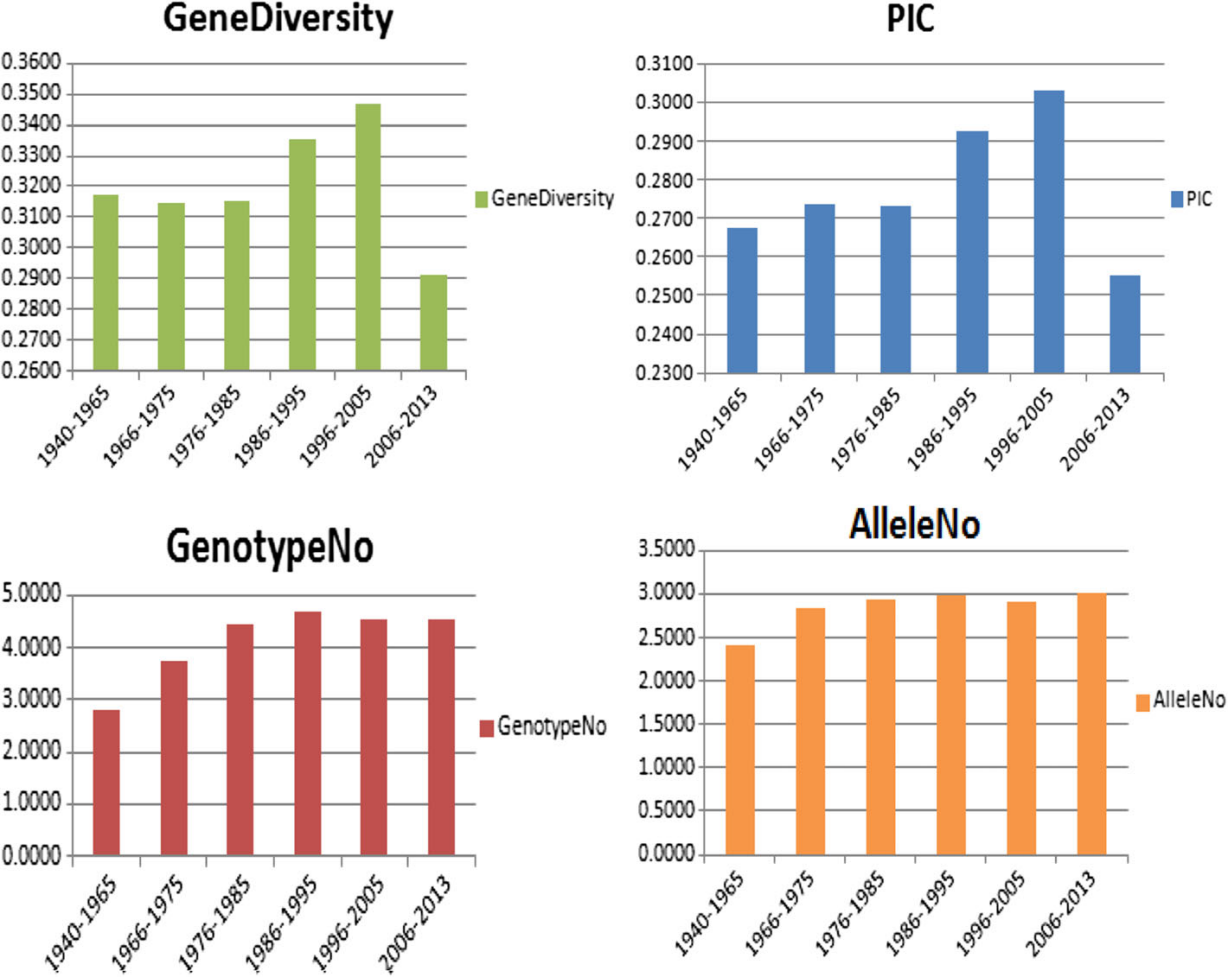
Graphs showing gene diversity, PIC, genotype no and allele number of the 729 rice varieties

All 729 rice varieties were divided into six different intervals on the basis of year of release and notification [1940–1965 (18 varieties), 1966–1975 (69), 1976–1985 (127), 1986–1995 (168), 1996–2005 (147), and 2006–2013 (136)]. Except 1940–1965, all intervals showed approximately comparable number of genotypes. The value of gene diversity, heterozygosity, major allele frequency and PIC of rice varieties falling in six time intervals were analyzed using the Power Marker. The mean value of gene diversity, heterozygosity, major allele frequency and PIC were plotted with years (time interval) on X axis and values of gene diversity, heterozygosity, major allele frequency and PIC on Y axis (Fig. 9).

## Additional files

Additional file 1: Table S1. List of rice varieties characterized using HvSSR markers along with their pedigree, IC number, year of release and traits. (XLSX 79 kb)
Additional file 2: Table S2. List of hybrid rice used in the present study with year of release and pedigree. (XLSX 10 kb)
Additional file 3: Figure S1. Detailed population structure of 729 rice varieties based on SSR data. (PDF 459 kb)

## Abbreviations

AMOVA: Analysis of molecular variance
HvSSR: Hyper variable simple sequence repeats
IC: Indigenous collection
Na: Number of alleles
NJ: Neighbor joining
PCoA: Principal coordinate analysis
PCR: Polymerase chain reaction
PIC: Polymorphic information content
pop: Population
SSR: Simple sequence repeats
