# Supplementary figures and images for: Genetic diversity trend in Indian rice varieties: an analysis using SSR markers
Source: BMC Genet. 2016 Sep 5;17:127. doi: 10.1186/s12863-016-0437-7 (PMC5011800; doi:10.1186/s12863-016-0437-7)

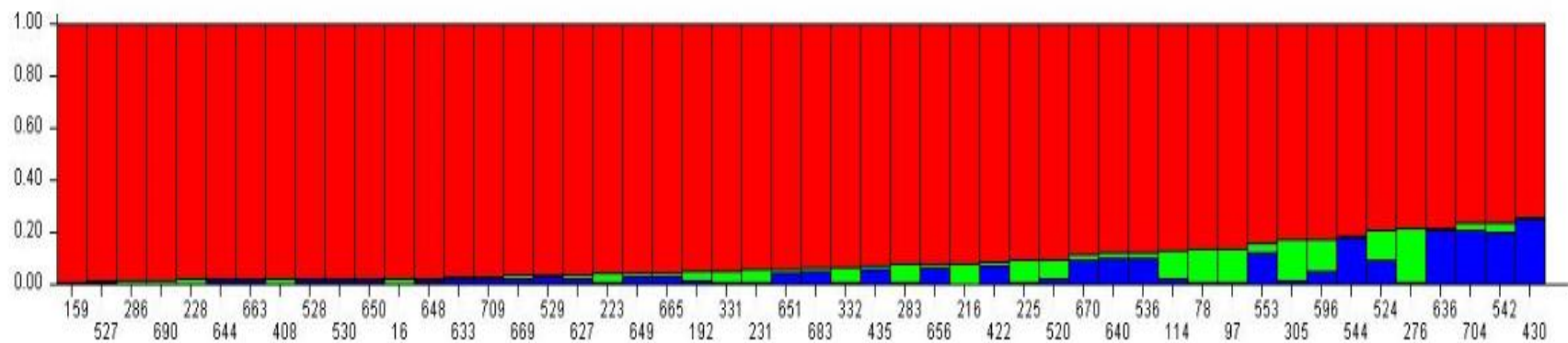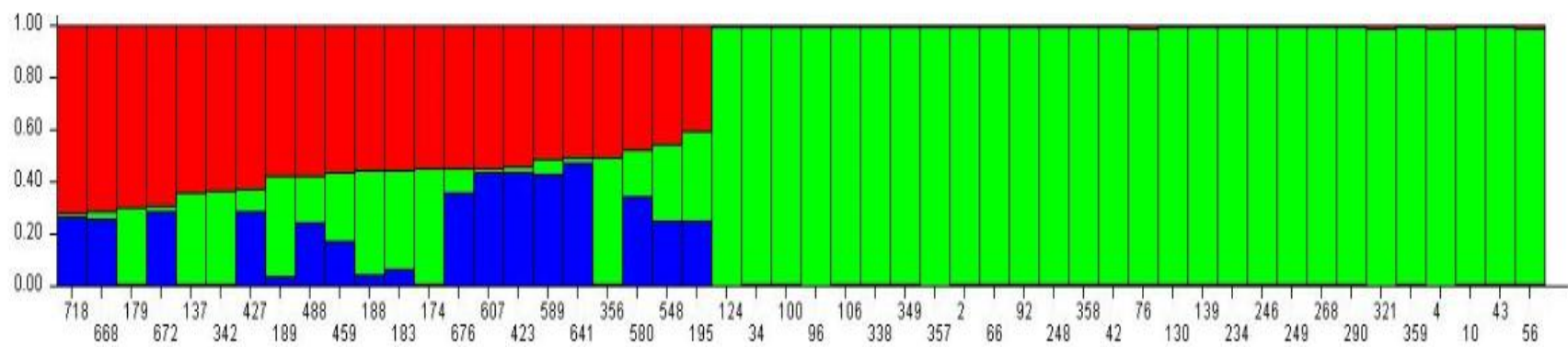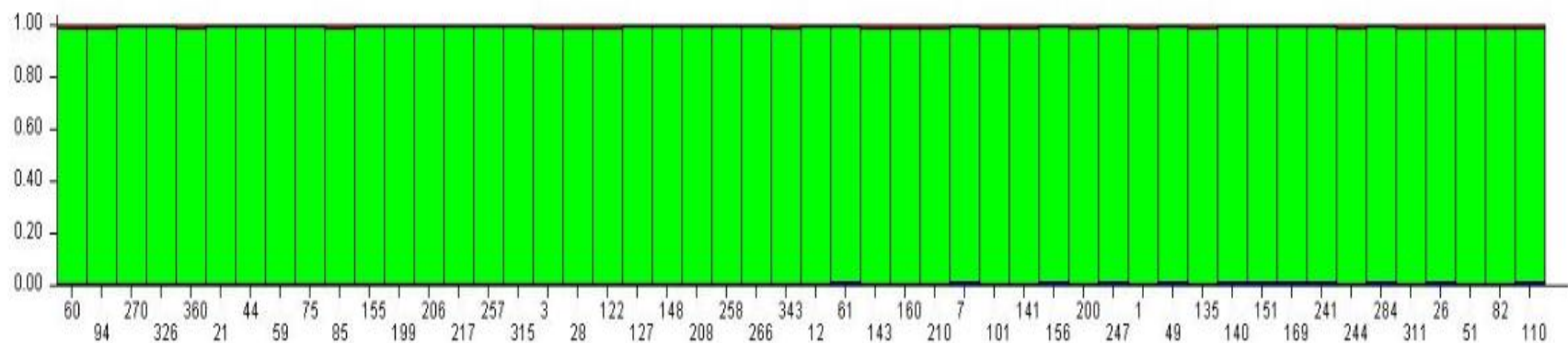

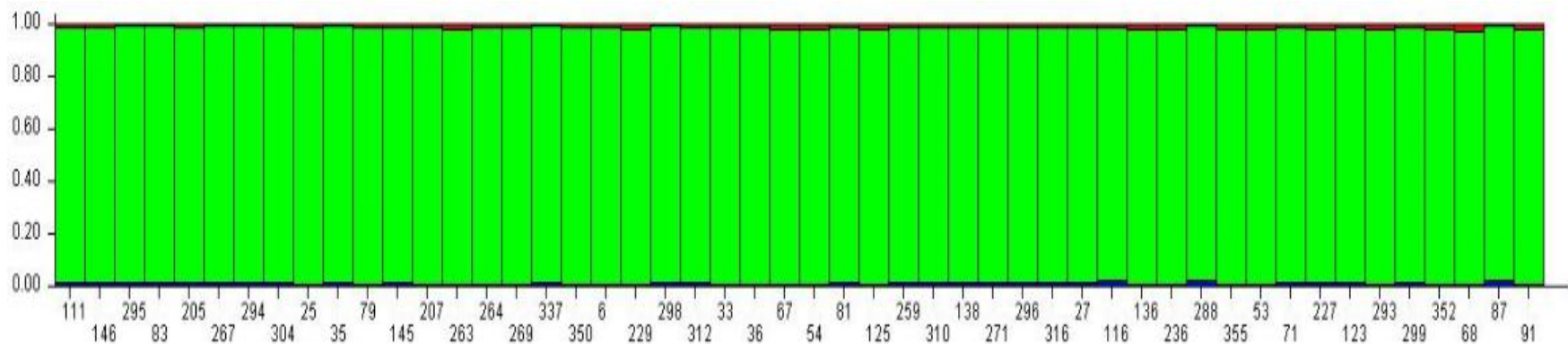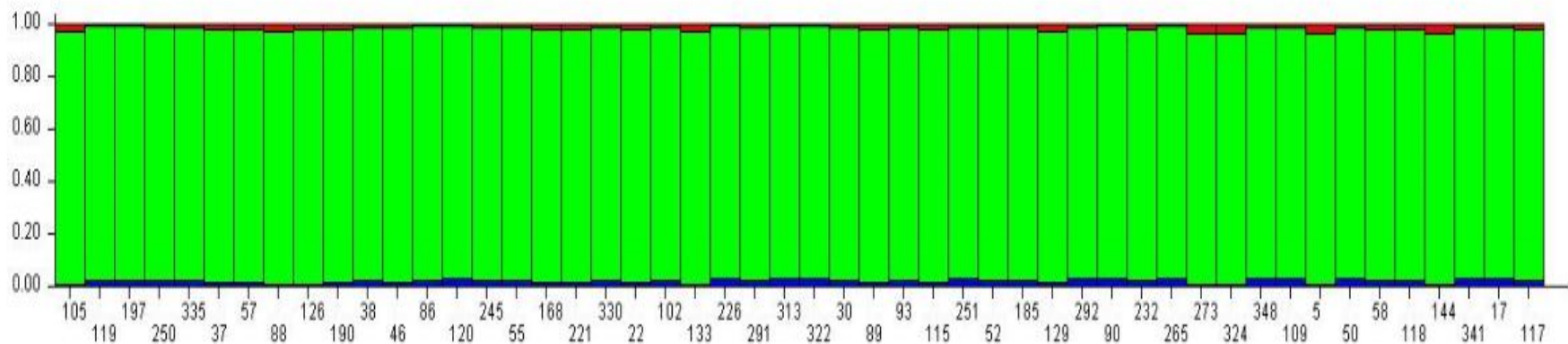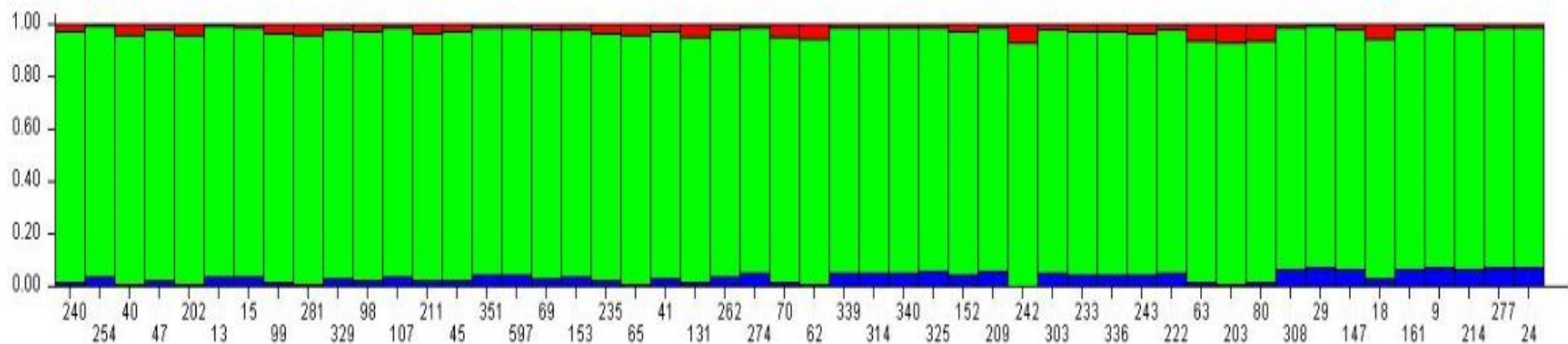

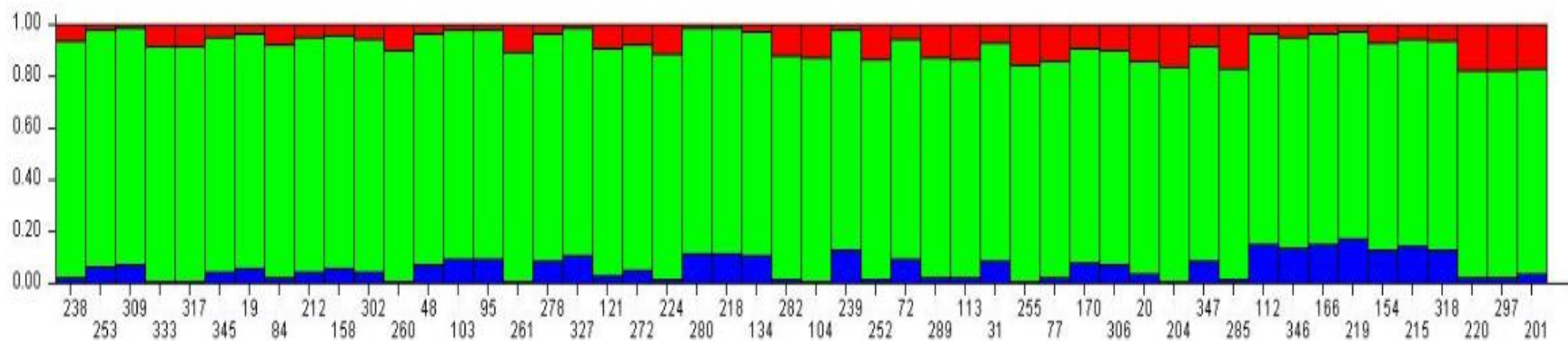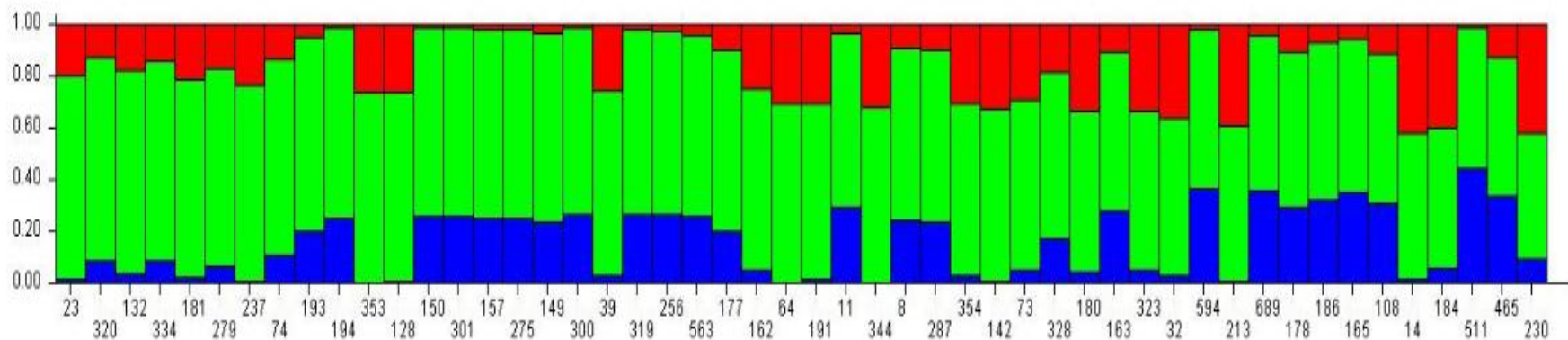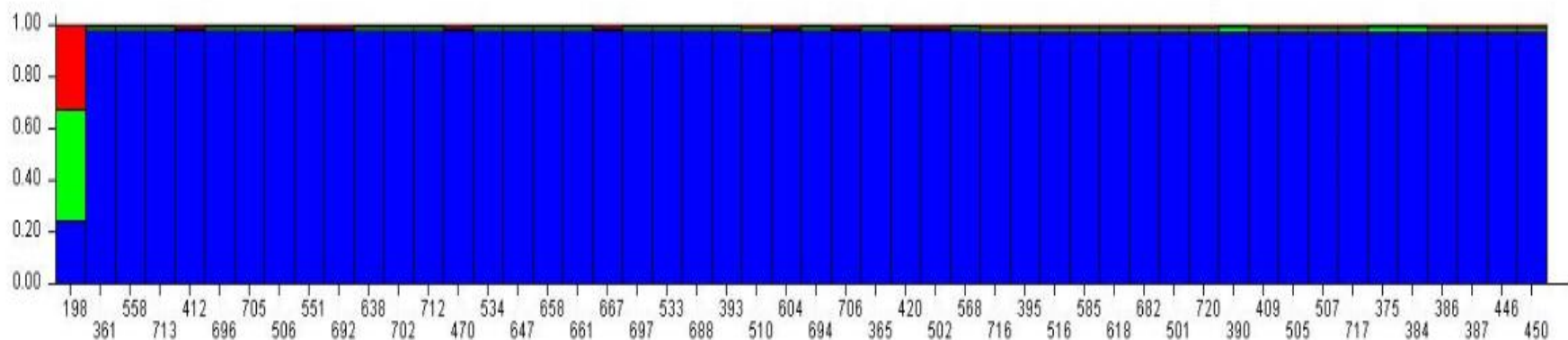

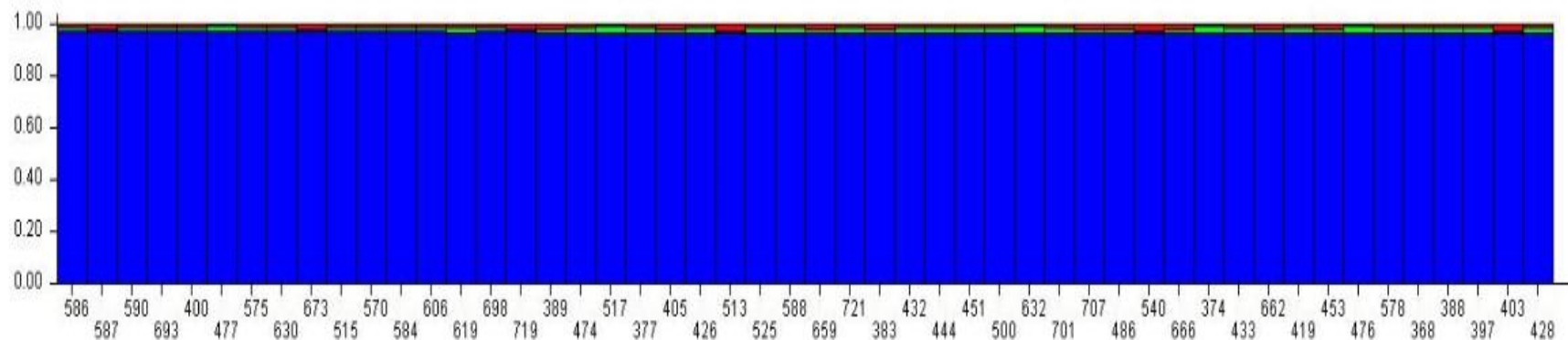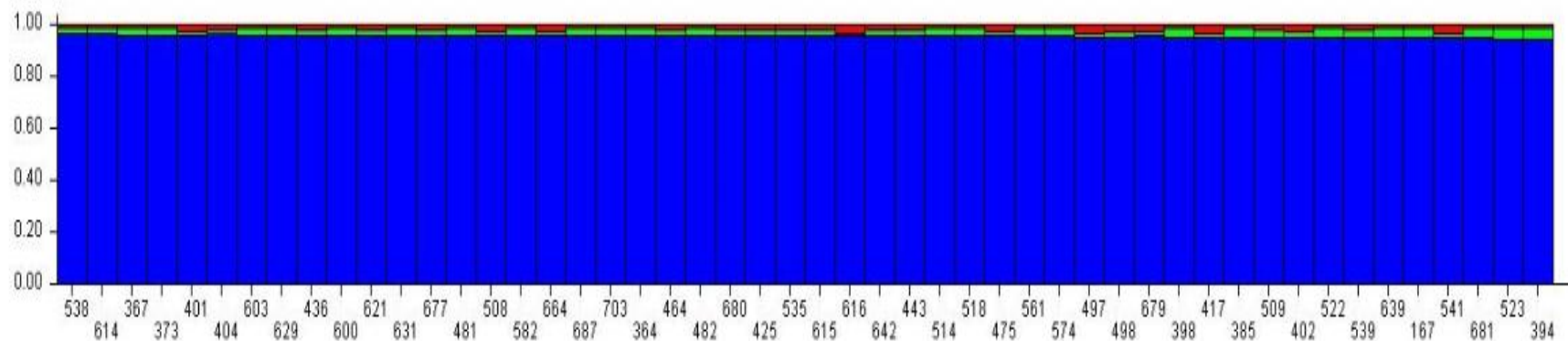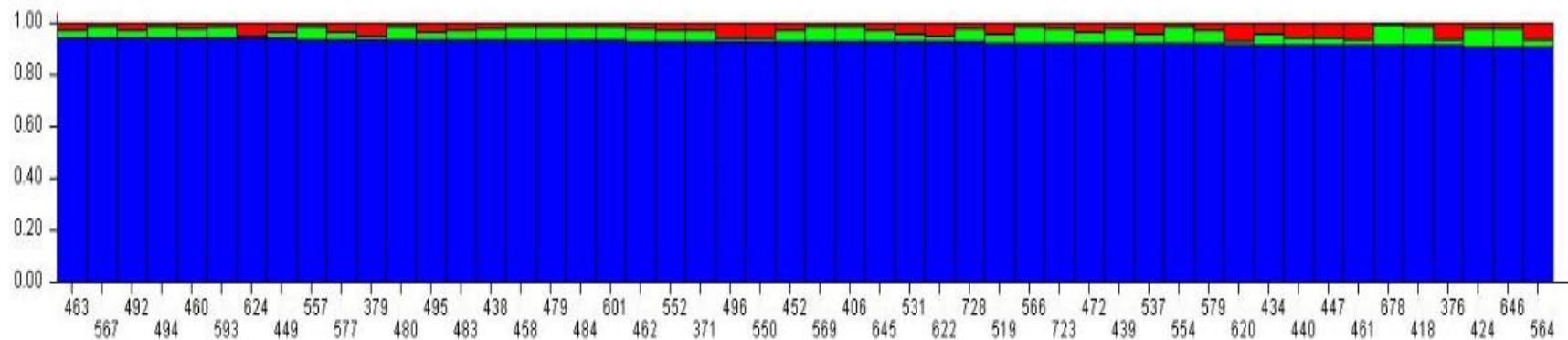

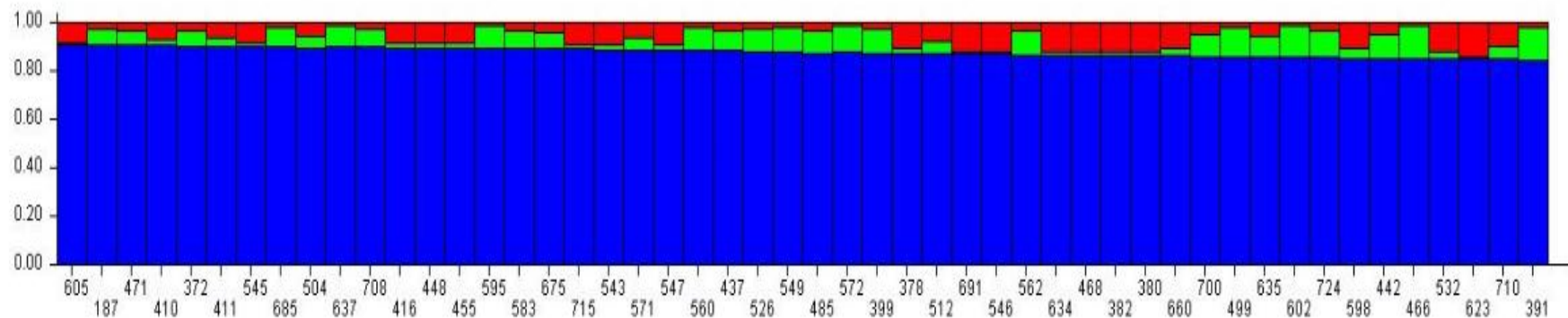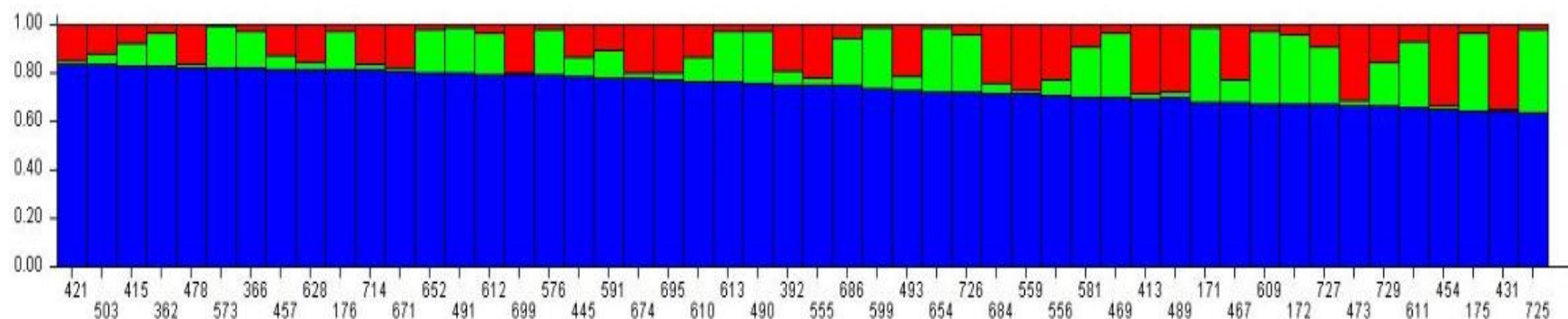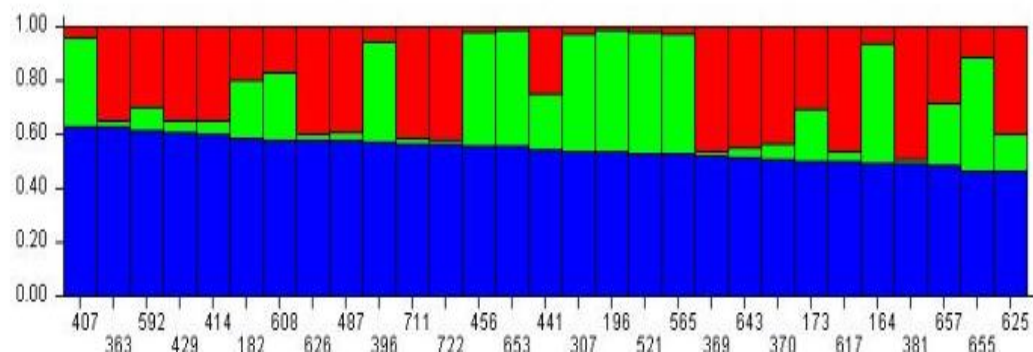

Supplement: Additional file 3: Figure S1. — Detailed population structure of 729 rice varieties based on SSR data. (PDF 459 kb) [file 12863_2016_437_MOESM3_ESM.pdf]
